# Structural Relationships between Highly Conserved Elements and Genes in Vertebrate Genomes

**DOI:** 10.1371/journal.pone.0003727

**Published:** 2008-11-14

**Authors:** Hong Sun, Geir Skogerbø, Zhen Wang, Wei Liu, Yixue Li

**Affiliations:** 1 Key Laboratory of Systems Biology, Shanghai Institutes for Biological Sciences, Chinese Academy of Sciences, Shanghai, China; 2 Biological Technologies, Wyeth Research, Cambridge, Massachusetts, United States of America; 3 Shanghai Center for Bioinformation Technology, Shanghai, China; 4 Bioinformatics Laboratory and National Laboratory of Biomacromolecules, Institute of Biophysics, Chinese Academy of Sciences, Beijing, China; 5 Zhongxin Biotechnology Shanghai Co. Ltd., Shanghai, China; University of California, Berkeley, United States of America

## Abstract

Large numbers of sequence elements have been identified to be highly conserved among vertebrate genomes. These highly conserved elements (HCEs) are often located in or around genes that are involved in transcription regulation and early development. They have been shown to be involved in *cis*-regulatory activities through both *in vivo* and additional computational studies. We have investigated the structural relationships between such elements and genes in six vertebrate genomes human, mouse, rat, chicken, zebrafish and tetraodon and detected several thousand cases of conserved HCE-gene associations, and also cases of HCEs with no common target genes. A few examples underscore the potential significance of our findings about several individual genes. We found that the conserved association between HCE/HCEs and gene/genes are not restricted to elements by their absolute distance on the genome. Notably, long-range associations were identified and the molecular functions of the associated genes do not show any particular overrepresentation of the functional categories previously reported. HCEs in close proximity are found to be linked with different set of gene/genes. The results reflect the highly complex correlation between HCEs and their putative target genes.

## Introduction

When the human genome became available, identification of all its functional genomic elements still remained difficult, and genomic comparisons have frequently been used to identify sequences with functional potential. Comparative genomics has highlighted the existence of an array of highly conserved non-protein coding regions in all vertebrates. Through the comparison of human and rodent genomes, more than 5,000 ultraconserved elements (UCEs) with 100 percent identity were found [1]. Hundreds of highly conserved non-coding elements (CNEs, UCRs) were also reported through long distance searching in the human and pufferfish genomes [2], [3]. A commonly observed characteristic of these highly conserved elements (HCEs; i.e. UCEs, CNEs and UCRs) is their strong tendency to occur in clusters along the chromosomes [1]–[3]. Comparative analysis has also shown that their relative order along the chromosomes is as conserved as that of coding genes in the mammalian genomes [4]. Among the mammals, the distances between pairs of HCEs are also more conserved compared to distances between protein coding genes. Thus, not only are their DNA sequences conserved but the relative positions of their loci are also stable [4].

Different studies have used slightly varying criteria to identify the highly conserved elements. Some studies included only non-coding genomic regions in their surveys, e.g. ultra-conserved regions (UCRs) [3] and highly conserved non-coding sequences (CNEs) [2], whereas others also included perfectly conserved exonic regions, e.g. UCEs [1]. Although it has been suggested that exonic UCEs represent a distinct subset in overlap with segmental duplications or copy number variants [5], additional studies indicated that exonic UCEs are also under multiple constraints with the enrichment of specific constituents of the cassettes in genes, e.g. 5′ UTR and 3′ UTR [5], which function in gene regulation. DNA coding sequences can also function as transcriptional regulatory elements [6], [7], exonic splicing enhancers [8], RNA secondary structure elements affecting mRNA stability, localization, or translation [9]. The potentially hidden regulatory signals within coding sequences have attracted considerable interest [10]. No satisfactory explanations for the extreme degree of sequence conservation of exonic UCEs have been suggested. Since 100 percent sequence identity on the DNA level is not required to maintain identical amino acid sequences, and thereby identical function of a protein, there are no a priori reasons to assume that exonic HCEs are principally different from HCEs at other genomic locations.

Though evolutionary analyses strongly support functional potential of these HCEs, most of their sequences' functional attributes remain unknown. Genes adjacent to the highly conserved non-coding elements are enriched in transcriptional and developmental functions [1]–[3], [11], [12]. There is a strong association between HCEs and the locations of genes encoding key regulators of development, and such association reflects a global genomic trend [3]. HCEs thus have frequently been suggested to function as *cis*-regulatory elements, and several HCEs have been tested as *cis*-regulatory modules of genes for early development [2]. Recently, a high propensity of extremely conserved human non-coding sequences have been shown to behave as transcriptional enhancers *in vivo*, and it has been proposed that the further 5,500 non-coding sequences conserved between humans and pufferfish may yield another new batch of gene enhancers [13].

There is no strong evidence for a direct role of genomic spacing in gene regulation at the present. Regulators located 1 Mb away from the target genes have been identified [14], [15]. A recent study showed the existence of long-range 3D interaction in genome, such as IgG loci [16]. The distance between HCEs and genes with up to five intervening genes is as conserved as the distance between HCEs and the nearest gene, raising the number of potential targets even higher, or, alternatively, suggesting that a considerable number of non-target genes may reside between an HCE and its target gene(s) [4]. Therefore, considerable distance ranges may exist between HCEs and their potential target genes. In a number of cases, regulatory modules controlling specific expression patterns of early development genes have been found to be conserved from fish to man [15], [17]. A set of associations between duplicated CNEs and their potential target genes has been predicted through a ‘paralogy mapping’ method [18]. Observation revealed that associations between HCEs and target genes were maintained in both copies after the whole genome duplication in teleosts with the loss of bystander genes, and that “genomic regulatory blocks” (GRBs) correspond to the long regions of conserved gene order across vertebrate genomes [19]. An HCE-gene association seems likely since there exists a general conservation of HCE position relative to their putative target gene [20]. If enhancer activity is the primary reason for the conserved sequence and the distance characteristics of the HCEs, then it is logical to assume that the HCE-target gene association should also be preserved during evolutionary history. However, it has not been shown that this principle applies to all (or the majority of) HCEs. We have therefore assembled three data sets from the previous studies [1]–[3] and undertaken a comparative analysis of the relationship between HCEs and their putative controlling genes across six different genomes.

## Results

A direct comparison element by element shows that two-thirds of the non-exonic UCEs [1] do not overlap HCEs from any of the two other data sets (Figure S1). The smallest data set of about 1,400 conserved non-coding elements (CNEs) [2] had the highest fraction of overlaps (∼80 percent), compared with about 50 percent for the set of ultraconserved regions (UCRs) [3]. We combined these three published data sets [1]–[3] to form an integrated data set consisting of 7,570 distinct highly conserved elements (HCEs) in the human genome, and used BLASTn with non-stringent parameters plus order and distance conservation criteria to locate all occurrences of the same HCEs in the mouse, rat, chicken, zebrafish and tetraodon genomes (Materials and Methods, Figure S2). More than 95 percent of 7,570 human HCEs could be anchored to the rodent genomes, 71 percent could be traced back to the chicken genome, and around 24 to 30 percent of the HCEs were found in fish.

Given the current hypothesis that HCEs are *cis*-regulatory elements (cREs) that have been conserved through vertebrate evolution, then presumably the *cis*-association to the regulated gene (e.g. “trans-dev”genes [1]–[3]) should also be conserved. This assumption would imply that for each HCE (or conserved HCE structure) there exists at least one gene that has remained in *cis*-configuration through the same span of evolutionary time that has conserved the sequence of the HCE. We therefore collected all HCEs that could be reliably identified in all six genomes, and subsequently identified all orthologous HCE-gene pairs that were located on the same chromosome in all species. A total of 947 HCEs were found in the human and other five query genomes (mouse, rat, chicken, zebrafish and tetraodon), and of these, 629 were associated with 331 different genes, resulting in 2,957 HCE-gene pairs common to all the six genomes. We further defined an HCE-gene linkage block (HGLB) as a set of one or more HCEs related to the same (or the same group of) genes, resulting in 85 six-way conserved HGLBs (Supplemental Results S1). We also defined an HCE/gene block as the same set of HCE(s)/gene(s) of an HGLB.

The proportion of conserved to all possible HCE-gene pairs shows various-degree reduction in the HCE-gene pairs' number under the constraint of different level of conservation (Methods S1, Table S1, Figure S3), the data reflects that using large evolutionary distances would significantly improve the signal to noise ratio. Under random assumption, the occurrence of these highly conserved associations is significantly rare (P = 1.68e-08, FDR = 1e-05; Table S2). We further supposed that the probability of finding a conserved HCE-gene pair is expected to be equal to the probability that both an HCE and a gene have not been separated by chromosomal rearrangement for a long period of evolutionary time. Chromosome recombination rates were used to estimate the probability of conserved HCE-gene pairs, and the probability decreases with the increase of HCE-gene distance as well (Methods S1, Figure S4).

**Figure 1:**
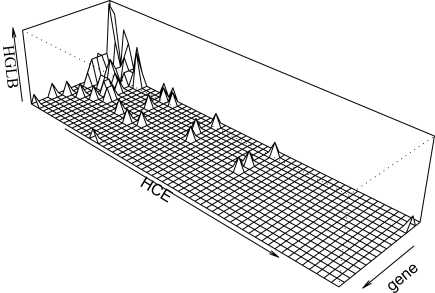
The number of HCEs and genes corresponding to the same HGLB. In a minority of HGLBs a single gene is associated with one or more HCEs. More often, several HCEs were associated with a number of common genes.

The number of HCEs and genes corresponding to the same HGLB presents a diverse picture (Figure 1). In a minority of HGLBs a single gene is associated with one or more HCEs. However, more commonly, several HCEs were associated with a number of common genes, with the more extreme cases being one HGLB constructed of 58 HCEs and four genes, and another including 17 genes linked with 16 HCEs. The class of single-gene HGLBs represents a genomic structure that allows for the potential identification of the target gene of one or a group of HCEs. Twenty-two HGLBs contain only a single gene, and are associated with 107 HCEs (Table 1). Contrary to some previous reports [1]–[3], no distinct bias was observed in the enrichment of molecular function of these 22 genes as assessed with GOToolBox tools [21] (Supplemental Results S2). The simplicity and significance of the 22 single-gene HGLBs may not be representative of the overall results. A few examples, however, underscore the potential significance of these genes.

**Table 1:** Twenty-two single-gene HGLBs.

Number of HCEs associated	Gene name	GeneOntology annotation
15	*PYGB*	phosphorylase activity; pyridoxal phosphate binding; transferase activity, transferring glycosyl groups
13	*TSHZ3*	metal ion binding; sequence-specific DNA binding; transcription factor activity; zinc ion binding
8	*CLORF125*	Not available
7	*ACVR2A*	ATP binding; contributes_to activin receptor activity; growth factor binding; inhibin beta-A binding; magnesium ion binding; manganese ion binding; nucleotide binding; protein self-association; receptor activity; transferase activity
7	*MMAA*	ATP binding; nucleoside-triphosphatase activity; nucleotide binding
7	*EBI2*	purinergic nucleotide receptor activity, G-protein coupled; receptor activity; rhodopsin-like receptor activity
6	*CTDP1*	DNA-directed RNA polymerase activity; hydrolase activity; phosphoprotein phosphatase activity
5	*PTCHD1*	hedgehog receptor activity
5	*KCNG3*	potassium ion binding; protein binding; voltage-gated ion channel activity; voltage-gated potassium channel activity
5	*VPS41*	metal ion binding; protein binding; zinc ion binding
4	*PTPRE*	hydrolase activity; receptor activity; transmembrane receptor protein tyrosine phosphatase activity
4	*UBR3*	metal ion binding; protein binding; zinc ion binding
4	*ZNF609*	metal ion binding; nucleic acid binding; zinc ion binding
4	*USP1*	cysteine-type endopeptidase activity; ubiquitin thiolesterase activity
2	*COQ3*	2-polyprenyl-6-methoxy-1,4-benzoquinone methyltransferase activity; O-methyltransferase activity; hexaprenyldihydroxybenzoate methyltransferase activity; transferase activity
2	*NDRG1*	protein binding
2	*USP9X*	cysteine-type endopeptidase activity; protein binding; ubiquitin thiolesterase activity
2	*BSX*	Not available
2	*CUGBP2*	RNA binding; nucleotide binding
1	*GLRB*	chloride ion binding; extracellular ligand-gated ion channel activity
1	*TMEM163*	Not available
1	*LRRC52*	protein binding

The functional description of associated genes is based on the Gene Ontology annotation.

**Figure 2:**
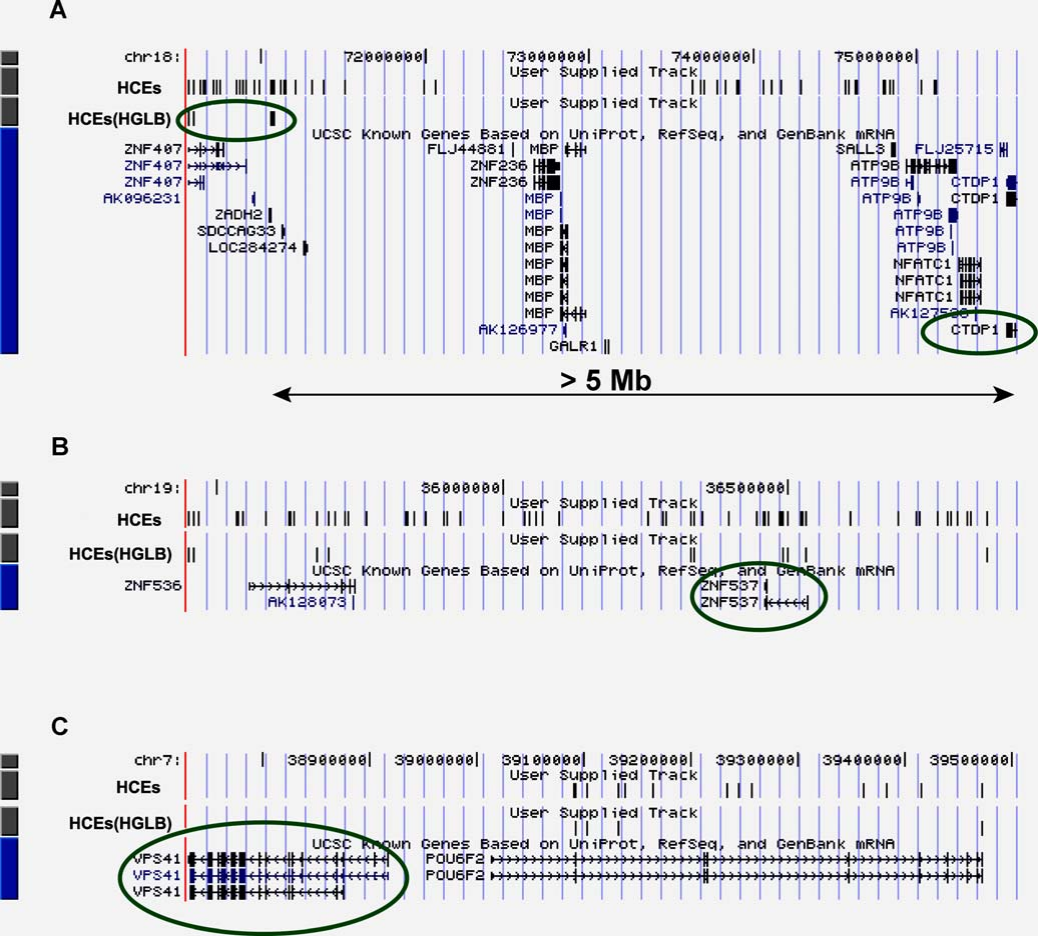
Human genomic environment of the three cases in which multi-HCEs are associated with only one gene. The three genes are all “trans-dev” associated, and labeled with surrounding oval. A: Six HCEs clustered in a 0.5 Mb region are linked with the disease-associated gene *CTDP1*. The *CTDP1* gene and the six HCEs are located more than 5 Mb apart. B: Thirteen HCEs are associated with the single gene *TSHZ3* (ZNF537). C: Five HCEs are linked with the gene *VPS41*.

One of the single-gene HGLB includes six HCEs clustered in a 0.5 Mb region on the human chromosome 18 and is associated with the gene *CTDP1*, which is located more than 5 Mb away from the nearest HCE (Figure 2A). *CTDP1* encodes a protein that interacts with the carboxy-terminus of the transcription initiation factor TFIIF, and a mutation in *CTDP1* has been identified as responsible for the Charcot-Marie-Tooth (CMT) syndrome [22]. In addition, a single-nucleotide substitution in an antisense Alu element in intron 6 of *CTDP1* causes congenital cataracts facial dysmorphism neuropathy (CCFDN) syndrome [23]. None of the other 14 human genes that are located in between *CTDP1* and the HCE block could be linked to these six HCEs. The HGLB overlaps completely a shorter genomic regulatory block (GRB) [19] which extends only 448 kb from *CTDP1*. Another single-gene HGLBs includes the gene “*TSHZ3*” and 13 HCEs that are scattered over a ∼1.4 Mb region on the human chromosome 19 (35.5 Mb–36.9 Mb, within which altogether 71 HCEs are embedded) (Figure 2B). *TSHZ3* contains one homeobox DNA binding domain and is a potential transcriptional regulator involved in developmental processes [24]. *TSHZ3* and its associated HCEs were also annotated as a gene regulatory unit by Kikuta *et al.* [19]. The third example of a single-gene HGLB contains five HCEs associated with the gene VPS41 which encodes a protein that has an important role in the segregation of intracellular molecules into distinct organelles (Figure 2C). Another gene, *POU6F2*, which encodes a transcription factor likely to be involved in early steps in the differentiation of amacrine and ganglion cells, is located near but is not associated with this HCE block.

We also examined the overlaps between HGLBs and recently identified genomic regulatory blocks (GRBs) [19], which were computationally predicted and experimentally verified. GRBs are chromosome segments with long-range cis-regulatory elements distributed over large areas in and around their target genes and surrounding non-homologous and functionally unrelated genes [19]. Fifty-two HGLBs overlap with 65 GRBs. HGLBs are commonly longer than the overlapping GRBs. In general, however, the percentage of overlapping length is small, with averages of 24.6 percent and 7.4 percent of the GRB and HGLB lengths, respectively (Table 2). Only one GRB is totally embedded within one single HGLB, and similarly, there is also only one HGLB embedded by a longer GRB. Of those HGLBs overlapping with GRBs, seven genes are common, which is not significantly rare against the whole human gene set background (Chi-squared test, data not shown). The data shows the validity of our approach and that these seven *cis* units are highly conserved. Still, it is also possible that quite a number of homology linked genes are not always HCEs' *cis*-regulatory targets.

**Table 2:** Percentage of overlapping length of HGLBs and GRBs (%).

	Min	Median	Mean	Max
Over the length of GRB	1.5	12.9	24.6	1
Over the length of HGLB	0.1	2.7	7.4	1

If a conserved cRE-gene association is the basis for the strong sequence conservation of the HCE, there should be at least one common target gene located on the same chromosome in all of the six genomes where an HCE is found. However, our method of conserved association analysis failed to detect a common target gene for 318 (33.6 percent) of the HCEs. Of these 318 HCEs, 92 are intergenic in all genomes. We further examined whether our failure to detect common targets was due to the incorrect identification of orthologous HCEs. Commonly observed characteristics of HCEs include a strong tendency to occur in clusters along the chromosomes [1]–[3] and to preserve relative orders [4]. Considering that HCEs common to all of the six genomes are far fewer than the total number of HCEs found in any query genome, we put this set of HCEs together with all of the other HCEs in the corresponding query genome. A comparison to the human genome showed that more than half (55.6 percent) of the 318 HCEs were located together with three or more other HCEs in all query genomes, and only 56 (17.6 percent) of the HCEs had a solitary location in one or more query genomes (Table S3). Furthermore, HCE clusters with more than 10 HCEs in preserved order were also found in all the query genomes, comprising 58 HCEs (Table S3). Thus, it is unlikely that a failure to detect associated genes for the majority of these HCEs is mainly due to incorrect annotation of HCEs in the query genomes. Though there is accumulating evidence in favor of *cis*-regulatory activity embedded in HCEs, our result suggests further investigation into the belief that HCEs are merely well-conserved cREs.

### Interlaced HGLBs

Several cases were observed where two or more HGLBs intersect each other in the human genome. What should be kept in mind is that HGLBs are defined corresponding to the unique set of homology linked HCE(s) and gene(s), and that both the associated HCE(s) and gene(s) are located on the same chromosome in all of the six genomes. A portion of HCEs, which were previously reported to be located in cluster [2], [3], are found to be divided into several sets associated with different HGLBs. All of these intriguing observations prompted us to look further into the genomic organization of HGLBs.

**Figure 3:**
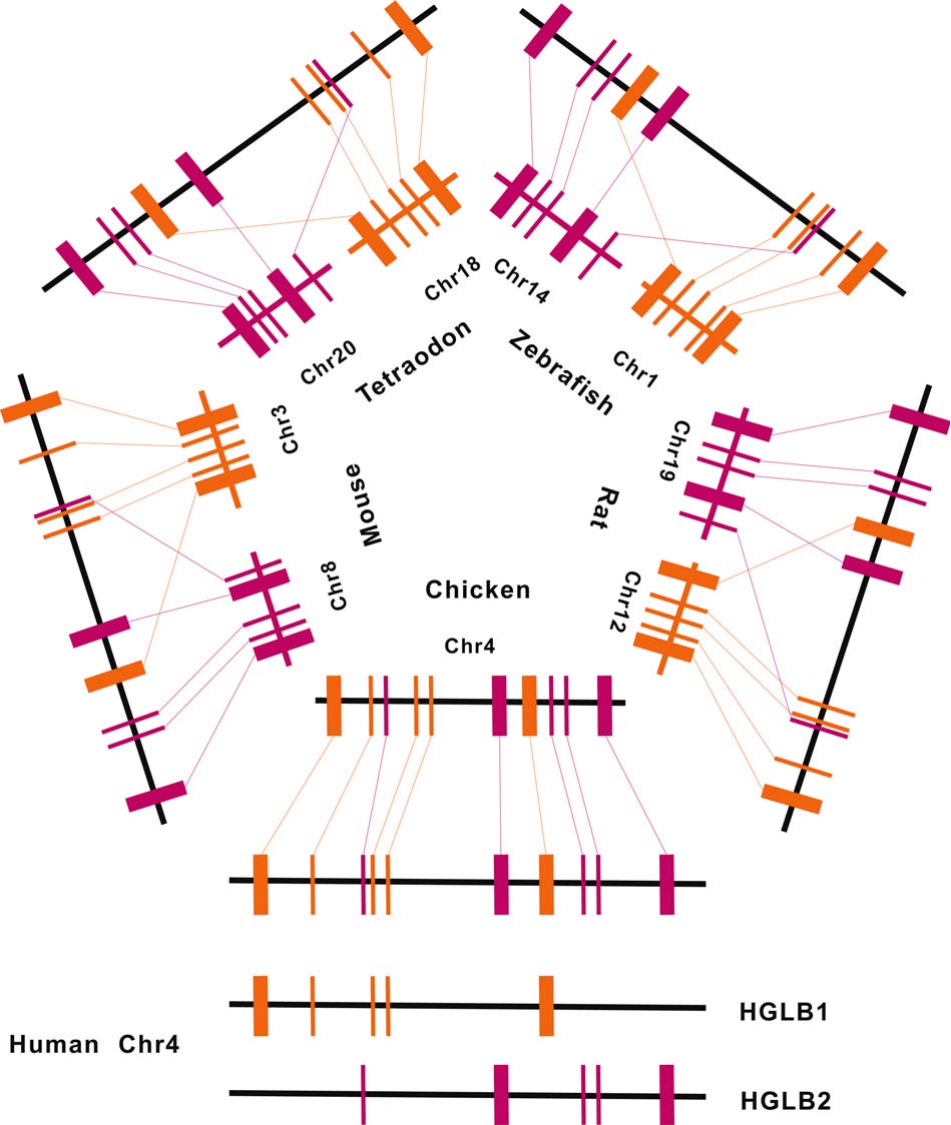
A sketch map of interlaced HGLBs. Two HGLBs interlaced each other on human chromosome 4. The two HGLBs reside in two different chromosomes in the mouse, rat, zebrafish and tetraodon genomes respectively. The conserved associations between HCEs and genes are not restricted to elements in relative proximity on the genome. HCEs belonging to the same cluster are divided into two HCE blocks, which are linked with two different sets of genes. (To compact the figure, several genes and HCEs are ignored and the size of the associated genomic region is not adjusted to the real scale. Rectangles represent genes while bars stand for HCEs. Lines link ortholgous sequence elements, and are labeled with the same color for the same HGLB block.)

We found 22 instances of intersecting HGLBs in the human genome, involving 54 of the total 85 HGLBs (64 percent). In most cases the associated HGLBs are located on different chromosomes of the fish genomes; however broken linkages were also observed between the mouse, rat and chicken genomes (Table S4). The conserved relationship between the HCE and 6-way orthologous gene observed from the interlaced HGLBs produced a complex picture. HCEs are not always linked with the nearest orthologous gene. In contrast, they are frequently found to be associated with genes far away (Figure 3). The individual HCEs within the same CNE/UCR cluster, which are originally defined according to their shorter inter distance, are not always linked with the same set of gene/genes (Figure 3). Likewise, intersecting HGLBs were observed in the non-human genomes (Table S5). The intuitive impression of this is that conserved associations between HCE/HCEs and gene/genes are not restricted to elements in relative proximity on the genome.

In general, one might expect that homology linked elements are necessarily located on the same synteny block, however we did not find sufficient evidence to support this hypothesis. Around 60 percent of the total 85 HGLBs were found intersecting with more than one four-way (human, mouse, rat, chicken) synteny block [25], and of these about two-thirds are covered by synteny blocks that are located on the same chromosome in all four species. In several cases, two or more synteny blocks overlap a single HGLB, some of them located on different chromosomes in at least one query genome (Table S6). Compared with the four-way synteny blocks derived from DNA/protein alignments among four species human, mouse, rat, and chicken HGLBs are also preserved in zebrafish and tetraodon, which is more stringent in the sense of evolutionary constraint. The average size of the four-way synteny blocks is 3.2 Mb and that of the HGLBs is 19.9 Mb in the human genome (Table S7). The overlapping analysis shows that HGLBs have no obvious relationship with the four-way synteny blocks. The way that syteny blocks are constructed influence the comparison with HGLBs. Genomic duplications, deletions, and rearrangements could happen at scales ranging from a single base to complete chromosomes. Large blocks of conserved synteny blocks are believed to be fragmented by small-scale evolutionary events, e.g. inversion, insertion/deletion, transposition and duplication [26]. We cannot exclude the possibility that to a larger extent, several long-range HGLBs were further fragmented by synteny blocks by large evolutionary events, and it is conceivable that chromosomal regions might contain specific “anchor points”, which have combined features of long-range chromatin modeling with *cis*-regulatory and/or other functions.

### The distances of HCE-gene pairs

Since the HCE-gene pairs identified by our method are not under *a priori* constraints such as mutual absolute distance or location in the same synteny blocks, *etc*, it is worth looking into whether the conserved associations we have identified would show biases in absolute distance between the HCE and gene, as well as the conservation of the distance of HCE-gene pairs. The HCE-gene pair distances were calculated as the distance between the midpoints of the HCE and the gene. A small fraction of HCEs overlapping with the associated genes (52 pairs) were excluded and the remaining 2,905 pairs were used for the distance analysis. Thus, excluding HCEs residing within genes, the minimum HCE-gene distance in the human genome is 1.7 Kb, and the median distance (6.2 Mb) is much shorter than the average (15.1 Mb) distance (Table 3). Though the skewed distance distribution underscores that most of the associations involve relatively closely located HCEs and genes (Figure S5), the fact that half of the HCE-gene pairs are more than 6.2 Mb apart suggests that a portion of HCEs may be related to (if to any) very distant genes.

**Table 3:** The distances of HCE-gene pairs in the human genome (Kb).

	Min	Median	Mean	Max
Distance of HCE-gene pairs	1.7	6,285	15,107	82,725

The distances were measured from midpoint to midpoint.

In order to analyze the effect of HCE-gene distance on the degree of distance conservation we divided the HCE-gene pairs into three groups according to the absolute distance. Of all HCE-gene pairs, 495 have a distance of less than 1 Mb, 884 are within 1–5 Mb of each other, and 1,526 are more than 5 Mb apart (Table S8). Relative distance differences (RDD) were calculated between the query and the human genomes. No significant difference in absolute value of RDD (|RDD|) was found among three groups in the comparisons between the human and mouse genomes; however, clear differences in |RDD| values were observed in the comparison between the human and the other four genomes. For the human-mouse comparison, the median |RDD| values for HCE-gene pairs with larger absolute distances were at a level similar to those with shorter absolute distances, indicating that the distances for the portion of HCE-gene pairs with larger absolute distances are also well conserved (Table S8).

The RDD values for HCE-gene pairs are distributed closer to zero than RDD values for gene-gene and exon-exon pairs in the comparison between the human and the rodent genomes, and the differences are statistically significant (Table S9, Figure S6). For the comparison of the non-mammalian genomes with the human genome, the distribution of RDD values for HCE-gene pairs show no distinct peak around zero. The RDD values cluster at negative values for both the human-chicken and human-tetraodon comparisons. This is in contrast to the RDD value distribution for the human-zebrafish comparison, where a two-peak profile was observed with one peak at positive value and the other at negative (Figure S6). The negative RDD values reflect the size difference between the human and zebrafish genomes, but there is no straight-forward explanation for the observation that a portion of HCE-gene pairs have more positive RDD values for the human and zebrafish comparison. It has been reported that at least 20 percentof zebrafish genes are present in duplicate [27], and it is possible that a fraction of the duplicate copies might have been lost, or that some missing duplicates may be present in the genome but not yet discovered. Using the InParanoid database [28] to test the potential duplication of human genes in the zebrafish genome, we found 1,577 human genes as potential duplicates. The genomic loci of 966 genes have been annotated based on the ensemble databases and 168 genes were found having at least two duplicates located on the same zebrafish chromosome. We thus suspected that HCE-gene pairs with positive RDD values for the human-zebrafish comparison may result from the assignment of the duplicated copy of HCE with the duplicated copy of the gene, with the “original” version of the gene (i.e. the one located closer to corresponding HCE in other genomes) having been lost in the zebrafish.

### HCE blocks, CNE/UCR clusters and distance conserved UCE blocks

An HCE block is defined as a region containing a set of HCEs associated with the same set of genes. The distribution of HCE block lengths in the human genome is highly skewed, with the median (0.2 Mb) being much shorter than the average (3.7 Mb) length (Table 4), indicating that some HCEs with long-distance interval are linked with the same set of genes. Even though a strong correlation exists between the HCE number and block length (Spearman's rho correlation coefficient = 0.87, p = 2.2e-16), quite a number of HCE blocks span rather long distances with relatively few HCEs (Figure S7). Half of the HCE blocks are made up of more than four HCEs (Table 4), with the most extreme case being a 1.8 Mb long HCE block composed of 58 HCEs associated with four genes.

**Table 4:** The length of HCE/gene blocks and the associated number of HCE(s)/gene(s).

		Min	Median	Mean	Max
HCE block	Length (Kb)	0.074	173	3,700	76,299
	Number of HCEs	1	4	7	58
Gene block	Length (Kb)	4	1,999	16,108	76,782
	Number of genes	1	2	3	17

Lengths (Kb) are measured based on the human genomic annotation.

In a previous study [4], we found stretches of UCEs [1] with strong distance conservation (|RDD|<0.15 [4] in comparisons between mammalian genomes). Sixty-eight HCE blocks overlap with 263 regions of consecutive UCE pairs with extremely conserved distances (|RDD|<0.15 [4]), and regions with highly conserved distances (|RDD|<0.15 [4]) cover more than half of the HCE blocks. We also tested the overlaps between HCE blocks and CNE/UCR clusters defined by two independent works of Sandelin *et al.* [3] and Woolfe *et al.* [2]. After converting the genomic coordinates of CNE and UCR clusters to version hg18, we obtained 165 CNE and 140 UCR clusters, respectively. Among the examples are that one single HCE block overlaps with more than one CNE/UCR cluster (Table S10). This can be explained by the fact that CNE and UCR clusters were defined mainly based on the density of the respective highly conserved elements along the chromosomes, whereas HCE blocks are not restricted by the physical distance between the HCEs. There are also several instances where one CNE/UCR cluster covers more than one HCE blocks (Table S10), due to the fact that the HCEs corresponding to the CNE/UCR cluster on a human chromosome are located on different chromosomes in at least one query genome. Around 12 percent of total HCEs are conserved throughout the six genomes. We further asked whether this selective relationship is limited to a small set of HCEs. In the pairwise comparisons between human and non-mammalian genomes, a high percentage of HCEs shared by the two genomes was found to be linked with orthologous genes. We also observed the complex conserved relationship in these two-way comparisons. Several CNE/UCR clusters divide into more than two two-way HCE blocks (Table S11), which indicated the selective linkage relationship was also presented in quite a number of HCE/HCEs.

These observations suggest it may be an oversimplification that HCEs (CNEs/UCRs) located in the relative vicinity on a human/mammalian chromosome represent one functional unit (or functional units associated with a single focus; e.g. a target gene). The data further indicates that HCE clusters may be composed of several functional units (or blocks of HCEs with different foci). Similarly, widely spaced HCEs may actually belong to a single functional unit (or have a common focus or foci), as also indicated by the wide spans of distance conservation between HCEs [4]

### Genes associated with HCE blocks

The average length of gene blocks is 16 Mb, with an average number of three genes per block (Table 4). The large average size of gene blocks indicates that some genes with long inter-distance are associated with the same set of HCEs. An additional finding that differentiates this analysis from the earlier reports [1]–[3] on HCE-gene relationships is that the molecular functions of the 331 genes found in the HGLBs do not show any particular overrepresentation of the functional categories previously associated with the genes in the vicinity of HCEs, and the only category with significant enrichment (p<0.01) was that of “protein binding” (Supplemental Results S3).

Possible associations between HCEs and their nearby genes have been analyzed by previous studies, which have found an over-representation of gene functional categories involving nucleic acid binding, transcription regulation and early development [1]–[3]. Of the (1,716) genes reported to be located nearby HCEs (UCRs) by Sandelin *et al* [3] (i.e. the closest three genes at any side of an UCR), only 72 were found in the dataset of 331 HCE-associated genes. We further looked into the number of intervening genes in between the associated HCE and gene. Previous studies have reported a number of instances of overlapping genes in eukaryotes [29]–[31], and thousands of overlapping genes were identified in the human genome [31]. Based on the human genome annotation, we counted overlapping genes as a single “gene”. On average, 132 intervening genes locate in between the associated HCE-gene pairs (Table S12, Figure S8). No significant increase in |RDD| for up to five intervening genes have been reported, thus raising the number of potential targets even further [4]. All these suggest that the relationship between HCEs and genes may be more complex than previously thought.

### Consistency of genomic location of HCEs

Genic (i.e. exonic and intronic) HCEs comprise large portions of HCEs, and they are expected to preserve to be located in the genic region of the same gene during the evolutionary process. To test whether HCEs are consistently associated with specific gene(s), we further analyzed the data with a particular focus on the genomic distribution of the HCEs. Previous studies (e.g. [1]) focused mainly on the human genomic annotation of HCEs. We have extended the analysis to the rodents and non-mammalian genomes. We consistently identify genomic locations of the HCEs in the six species: human, mouse, rat, chicken, zebrafish and tetraodon. Of the 7,570 HCEs, 947 are shared by all six species and of these only 33 percent (312 HCEs) are consistently exonic, intronic or intergenic across these six species (Table 5). The remaining 635 HCEs show variable genomic locations from one genome to another. A total of 86 HCEs preserve the same genic context in all six of the genomes, and the genes associated with the same HCE(s) are found to be homologous among two or more species. More than 65 percent of HCEs are located in the genic region in one set of genome(s) but in the intergenic region of other sets of genome(s). It is expected that HCE genomic location should be more conserved for the comparison between human and rodent than between human and non-mammalian species due to the relatively shorter evolutionary distance. Interestingly, some of the HCEs that have preserved the same type of genomic locations in both human and non-mammalian genomes have a different genomic location in rodents. A total of 175 HCEs are genic (exonic or intronic) among human and the three non-mammalian genomes, but only about half are genic in the rodents.

**Table 5:** The number of HCEs with consistent genomic location in the six genomes.

	exonic	intronic	intergenic	genic
Number of HCEs	35	34	243	86
Percentage (%)	3.7	3.6	25.7	9.1

One possible interpretation for the lack of consistency in HCEs' genomic location is the imprecise genomic annotation; however, it is difficult to believe that such an assumption would be true in so many cases. Whereas it has been suggested that the human exonic HCEs represent a distinct subset [5], our data does not exclude the possibility that an HCE harbored by a gene is not necessarily its “associated” gene, or that there may not be a gene specifically associated with an HCE. The data also suggests that HCEs' genomic context or the local environment surrounding them might not always restrict their potential function.

## Discussion

If genes conserved across species are also conserved at the level of their transcriptional regulation, then there presumably exists a conserved *cis*-regulatory organization of HCEs and their target genes. As expected from this premise, a percentage of conserved HCE-gene associations was identified with complex relationships. Both long distance and relatively closely related associations between HCEs and genes were identified. No significant increase was found in |RDD| values for HCE-gene pairs with large absolute distances. Furthermore, quite a number of conserved HCE-gene associations were found with a large number of intervening genes. Genes over-represented in the vicinity of HCEs show a significant enrichment in certain functional categories involving transcription regulation and early development, as reported previously [1]–[3]. Surprisingly, genes linked with HCE(s) do not display any strong enrichment for particular molecular functions. The extreme sequence conservation of HCEs suggests that these elements play vital roles for their host; however, deletion of HCEs failed to reveal any critical abnormalities and showed an apparent lack of association to nearby genes [32]. All the facts suggest that the relationship between HCEs and genes may be more complex than previously supposed.

Not all HCEs shared by the six genomes have gene(s) with conserved association. One intuitive interpretation of this observation would be that these HCEs do not have *cis*-regulatory function, or, alternatively, that the same HCEs regulate different genes in different species. Genes associated with HCEs have been reported with strong statistically significant enrichment for certain functional categories, including early embryo development and other transcription factors [1]–[3]. If their function is as important as the extreme degree of sequence conservation would indicate, inconsistent regulation of target genes might cause dramatic change in vertebrate development with potentially profound effects. The inconsistency of genomic location of HCEs makes it less likely that *cis*-regulation is their major role [33]. Although neither distance conservation nor homology analyses of conserved associations are sufficient or ideal to identify all potential target genes, our results strongly suggest that the hypothesis that the majority of HCEs are *cis*-regulatory elements for a distinct set of genes still needs to be treated with care. Suggestions can be made that HCEs essentially belong to the same population of sequence elements, as shown by the same extent of HCE-HCE distance conservation and HCE depletion among segmental duplications and copy number variants [5]. A strong suggestion has been put forward by a recent study that HCEs function as “counting units” since they are both conserved and unique [5]. Our data oppugn the merely *cis*-regulatory modules of HCEs, yet it does not exclude the possibility that participation of HCEs in other function(s) is accompanied with the involvement of their enhancer-like activities. Our results not only broaden our understanding of HCEs' function beyond the notion that HCEs are merely well-conserved cREs, but also give us a few clues to understanding other aspects of HCEs. A notable peculiarity is their independence, which can be inferred from our homology analysis. The inconsistency in genomic locations suggests that their potential function is not confined by the local genomic context, which means not being confined by the genes harboring it, though there are other constraints to limit their location, e.g., relative distance conservation [4]. On the other hand, it may also suggest that at least one of their potential functions, if it exists, is not restricted to coding activity.

The potential functional association between HCE(s) and gene(s) is not only complicated by the existence of long distance linked HCE-gene pairs, but also by the observation of the independence from HCEs' genomic environment. Some highly conserved HCE-gene pairs have supporting information of genomic regulatory association from other works [19], which support our method for finding phylogeneticaly conserved *cis*-regulatory modules or other functional linkage. The flexible genomic location and linkage with genes do not necessarily indicate that HCEs are irrelevant with each other or with gene(s). Though it is difficult to pin-point their exact function immediately, the highly conserved associations do suggest evolutionary constraint on these connections. Multiple alignments of the species under comparison would allow for the precise identification of conserved HCEs among the genomes, and allow for more detailed homology analysis [34]–[36]. It is to be hoped that deeper analysis of sequences homology/conservation between sequenced genomes will produce additional genetic elements whose positions can be identified with reasonable certainty, so that association conservation can studied for a larger part of the genomes.

The results of the homology analysis of conserved association between HCEs and genes may be influenced, to some extent, by the highly complicated genome structure of vertebrate genomes. As much as 15 percent of human genes are duplicated with segmental duplications covering 5.2 percent of the genome [37]. Comparative study suggests that a genome duplication event has happened in the ancestry of teleost fish [27]. This high degree of duplication in addition to other genomic rearrangements makes it difficult to distinguish orthologous genes from paralogous genes and pseudogenes, and orthologous non-coding sequences from paralogous sequences. Failure to detect some potentially conserved HCE-gene associations may be due to the lack of precise and complete genome annotation. It is also difficult to eliminate the possibility that some HCEs and genes locate on the same chromosome across the six species without having any functional association. More extensive genome annotation of the regions may reveal more associations between linked HCEs and genes.

## Materials and Methods

### Data

Genome sequences were downloaded from UCSC GoldenPath database for the six species: human (hg18), mouse (mm7), rat (rn4), chicken (galGal2), zebrafish (danRer3) and tetraodon (tetNrg1). UCE [1] and CNE [2] dataset were obtained from the respective authors. The UCR [3] dataset was obtained from http://mordor.cgb.ki.se/cgi-bin/SCRbrowse/c. The collections of annotated genes for all these species were downloaded from UCSC GoldenPath database (http://hgdownload.cse.ucsc.edu/goldenPath). Collections of pair wise orthologous groups between human and other genomes were downloaded from InParanoid database [28]. We obtained four-way human-mouse-rat-chicken synteny blocks from Bourque *et al* [25], and genomic regulatory regions (GRB) from Kikuta *et al.* [19] .

The three datasets of conserved elements were integrated together. Using the human genome as reference, we extended physical loci to the most remote start/end position of those elements which have intersection with each other, and we obtained 7,570 highly conserved elements (HCEs) without overlap.

### Assignment of unique homologous HCE hits

HCEs were aligned against genomes using BLASTn with non-stringent parameters (mismatch penalty −1, gap open penalty 1, word size 9, and soft masking). Only those hits with e-values less than 10^−5^ were kept for further analysis.

In cases where some HCEs have multi-alignment hits and some have no BLASTn hit in the query genome, two hits were looked as one pair according to the query genome, if there are less than two other HCEs located in between the two consecutive HCEs in the human and other species' genomes. RDD [4] values were calculated to measure the conservation of distance between the HCEs pairs. The pairs which were unique in the non-mammal genome were kept, and were divided into three categories according to their linkage with other HCE pairs or associated orthologous genes. For the HCEs with multi-BLASTn hits pairs, we treat them as the corresponding HCEs in the non-mammal genomes on the condition of linkage with other HCE pairs or orthologous genes. Because HCEs tend to be located in clusters, linkage condition of HCE pairs is the first screening step. Thus, the corresponding |RDD| value might not be the minimum. If there were no existing linkage, the two consecutive HCEs with minimum |RDD| value were kept and thus position with the corresponding HCEs in the query genome.

### Assignment of conserved HCE and gene pairs

Long-range regulation have been identified [14], [15], therefore we introduced no constraint on the absolute distance between HCEs and their putative target gene(s) except for a loose criteria to be on the same chromosome, which is the characteristic of *cis* action. An HCE and a gene were regarded as an HCE-gene pair if they were found on the same chromosome in the genome. Various works have been demonstrated the interspecies conservation of regulatory modules [15], [17]–[19], thus conservation of pairing was added for a further screening. An HCE-gene pair was considered to be conserved if it was found in all species investigated.

We analyzed conserved associations between HCEs and genes among the human, mouse, rat, chicken, zebrafish and tetraodon genomes.

### Statistical analysis of finding highly conserved HCE-gene pair

The null hypothesis is that HCE-gene pairs are randomly linked in all of the six species examined. Given a species i, the probability of finding a random HCE-gene pair is 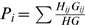, where H, G are the total number of HCEs and genes conserved in all the species examined; and H_ij_, G_ij_ are the corresponding number on chromosome j. Under random match assumption, the probability of the observation in all the six species is 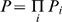, which can be treated as p-value for a HCE-gene pair under the null hypothesis. Of all possible HCE-gene pairs, the false positive rate (FDR) is H*G*P/R [38], R is the number of real findings.

### Calculation of distance differences

We calculated RDD values [4] to measure the relative distance difference between pairs of genomic elements, RDD = (*d*_q_−*d*_h_)/[(*d*_q_+*d*_h_)/2]; where *d*_q_ and *d*_h_ being the distance between the mid-points of two sequence element pairs in the query (non-human) and human genomes, respectively.

### Gene ontology annotation analysis

We compared gene ontology (GO) annotations of genes associated with the HCE-gene pairs in the human genome against the background of all annotated human genes, using the hypergeometric distribution test to calculate P-vales and adjusted for the occurrence of false positives using the Bonferroni correction method [39]. GO molecular function analysis was performed by using the GOToolBox [21]. Statistical analyses were carried out using the R language and software [40].

## Supporting Information

Methods S1 Supplementary Methods
(0.02 MB DOC)

Supplemental Results S1 HGLBs in the human genome (hg18)
(0.03 MB TXT)

Supplemental Results S2 The enrichment of molecular function of 22 genes.
(0.00 MB TXT)

Supplemental Results S3 The enrichment of molecular function of 331 genes
(0.02 MB TXT)

Table S1 The number of HCE-gene pairs decreases when species is added for the comparison.
(0.03 MB DOC)

Table S2 Statistics of finding an HCE-gene pair.
(0.02 MB DOC)

Table S3 Statistics of the linkage relationship between 318 HCEs and all of the HCEs identified in the query genomes.
(0.03 MB DOC)

Table S4 The number of cases of HGLBs interlaced in the human genome but located on different chromosomes in other species.
(0.03 MB DOC)

Table S5 The number of HGLBs involved in the intersections in different vertebrate genomes.
(0.03 MB DOC)

Table S6 The number of HGLBs overlapped with 4-way synteny blocks in the human genome.
(0.04 MB DOC)

Table S7 Sizes of HGLBs and 4-way syteny blocks in the human genome (Kb).
(0.03 MB DOC)

Table S8 Absolute relative distance differences (|RDD|s) of HCE-HCE pairs and HCE-gene pairs.
(0.04 MB DOC)

Table S9 Difference of |RDD| values for different pair wise elements.
(0.03 MB DOC)

Table S10 Overlapping between HCE blocks and CNE/UCR clusters.
(0.06 MB DOC)

Table S11 Summary statistics of HCE-gene pairs in the pair-wise comparisons between human and three non-mammal genomes.
(0.04 MB DOC)

Table S12 Percentage of genes associated with HCEs over total genes in the overall genomic region covered by HGLBs, and the number of intervening “genes” in between each HCE-gene pair in the human genome.
(0.03 MB DOC)

Figure S1 Overlapping between HCEs from different data sets. The figure shows UCRs (red), CNEs (green) and UCEs (blue) with at least partial (more than 1 bp) overlapping in the human genome. The data from the three studies were derived from three different versions of the human genome sequence, and had to be mapped onto a common version for comparison. Thus, the number of total HCE for the two first datasets differs slightly from the figures published by the original studies.
(0.52 MB TIF)

Figure S2 The flowchart to assign unique homologous HCE hits in the five query genomes.
(0.01 MB TIF)

Figure S3 Number of conserved HCE-gene pairs at different conservation level. (HM: in the human-mouse comparison; HMR: in the human-mouse-rat comparison etc. H stands for human, M for mouse, R for rat, C for chicken, Z for zebrafish and T for tetraodon)
(1.26 MB TIF)

Figure S4 Plot of the probability of finding conserved HCE-gene pairs and the distances of the HCE-gene pairs in the human genome.
(0.01 MB TIF)

Figure S5 Histogram of distances of the 2905 HCE-gene pairs in the human genome.
(0.01 MB TIF)

Figure S6 RDD distribution of four sets of data (HCE-HCE, HCE-gene, Gene-Gene and Exon-Exon).
(0.16 MB TIF)

Figure S7 Plot of the number of associated HCEs and length of HCE blocks. (Spearman's rho correlation coefficient = 0.87, p value = 2.2e-16)
(0.03 MB TIF)

Figure S8 Histogram of the number of genes intervening HCE-gene pairs based on the human genome annotation. The number of genes overlapping in their genomic loci was counted as one.
(0.96 MB TIF)
