# Supplementary material for: Structural Relationships between Highly Conserved Elements and Genes in Vertebrate Genomes
Source: PLoS One. 2008 Nov 14;3(11):e3727. doi: 10.1371/journal.pone.0003727 (PMC2579482; doi:10.1371/journal.pone.0003727)
Supplement: Methods S1 — Supplementary Methods (0.02 MB DOC) [file pone.0003727.s001.doc]

**Methods S1**

The total number of HCE-gene pairs on the same chromosome within one species is $N=\sum_{i} H_{i}G_{i}$ , where H_i_, G_i_ is the number of HCEs and Genes on chromosome i. For each species, the number of HCE-gene pairs is very large before adding the constraint of interspecies conservation. We calculated the proportion of conserved to all possible HCE-gene pairs and the data shows various-degree reduction in the HCE-gene pairs’ number under the constraint of different level of conservation (Table S1, Figure S3). Of the HCE-gene pairs shared by human and mouse, about 25 percent were reduced after the inclusion of rat for comparison, whereas the inclusion of chicken leads to a more than 70 percent decrease. The data reflects that using large evolutionary distances would significantly improve the signal to noise ratio. The proportion of conserved to all possible HCE-gene pairs shows various-degree reduction in numbers under the constraint of different levels of conservation. The statistical analysis of locating these highly conserved associations in the six genomes shows the significance of our finding (p_value = 1.68e-08, FDR = 1e-05; Table S2).

The number of HCE-gene pairs decreases with increasing distance with a power law profile (Figure S5), which indicates that long distance association are more easily separated by chromosomal rearrangement. The probability of finding a conserved HCE-gene pair is expected to be equal to the probability that both an HCE and a gene have not been separated by chromosomal rearrangement for a long period of evolutionary time. Chromosome recombination rate and r = 0.5*(1-exp(-2*x)) mapping function was used to estimate the probability of conserved HCE-gene pairs, which have not been separated by chromosomal rearrangement. The chromosome wide recombination rate for human, mouse and rat was obtained from Jensen-Seaman et al [1]. Since a chromosome wide recombination rate was not readily available for chicken, zebrafish and tetraodon, the average rate across the corresponding whole genome was used. Given a species i, the probability of conserved HCE-gene pairs is $P_{i}=\left(1-r_{i}\right)$, r is the chromosome recombination probability. The combined observation in all the six species is $P=\prod_{i} p_{i}=\prod_{i} \left(1-r_{i}\right)$, and the probability decreases with the increase of HCE-gene distance as well (Figure S4).
