# Supplementary material for: Structural Relationships between Highly Conserved Elements and Genes in Vertebrate Genomes
Source: PLoS One. 2008 Nov 14;3(11):e3727. doi: 10.1371/journal.pone.0003727 (PMC2579482; doi:10.1371/journal.pone.0003727)
Supplement: Table S1 — The number of HCE-gene pairs decreases when species is added for the comparison. (0.03 MB DOC) [file pone.0003727.s005.doc]

| Number of HCE-gene pairs | HM | HMR | HMRC | HMRCZ | HMRCZT |
| --- | --- | --- | --- | --- | --- |
|  | 1,897,160 | 1,414,386 | 384,843 | 10,477 | 2,957 |
| Percentage of reduction  (%) | 25.4 | |  |  |  |
|  |  | 72.8 | |  |  |
|  |  |  | 97.3 | |  |
|  |  |  |  | 71.8 | |

HM: in the human-mouse comparison; HMR: in the human-mouse-rat comparison etc. H stands for human, M for mouse, R for rat, C for chicken, Z for zebrafish and T for tetraodon.
