# Supplementary material for: Structural Relationships between Highly Conserved Elements and Genes in Vertebrate Genomes
Source: PLoS One. 2008 Nov 14;3(11):e3727. doi: 10.1371/journal.pone.0003727 (PMC2579482; doi:10.1371/journal.pone.0003727)
Supplement: Table S2 — Statistics of finding an HCE-gene pair. (0.02 MB DOC) [file pone.0003727.s006.doc]

| Species | Human | Mouse | Rat | Chicken | Zebrafish | Tetraodon |
| --- | --- | --- | --- | --- | --- | --- |
| P value | 0.050 | 0.054 | 0.056 | 0.060 | 0.035 | 0.053 |

The probability of finding a HCE-gene pair is $P_{i}=\sum_{j} \frac{H_{ij}G_{ij}}{HG}$for species i; H, G are the total number of HCEs and genes conserved in all the species examined; and H_ij_, G_ij_ are the corresponding numbers on chromosome j. (The probability of finding a conserved HCE-gene pair in all six species is $P=\prod_{i} P_{i}$≈ 1.68e-08, FDR = H*G*P/R ≈1e-05)
