# Supplementary material for: Structural Relationships between Highly Conserved Elements and Genes in Vertebrate Genomes
Source: PLoS One. 2008 Nov 14;3(11):e3727. doi: 10.1371/journal.pone.0003727 (PMC2579482; doi:10.1371/journal.pone.0003727)
Supplement: Table S3 — Statistics of the linkage relationship between 318 HCEs and all of the HCEs identified in the query genomes. (0.03 MB DOC) [file pone.0003727.s007.doc]

| Number of HCEs ordered together  in all the six genomes (≥) | | 2 | 3 | 4 | 5 | 6 | 7 | 8 | 9 | 10 |
| --- | --- | --- | --- | --- | --- | --- | --- | --- | --- | --- |
| HCEs with no common target gene(s) | Number | 262 | 177 | 142 | 118 | 107 | 94 | 89 | 66 | 58 |
|  | Percentage (%) | 82.4 | 55.6 | 44.6 | 37.1 | 33.6 | 29.5 | 27.9 | 20.7 | 18.2 |

A total of 318 HCEs have no target gene(s) with conserved association in all of the six genomes. Compared with the human genome, only 17.6 percent of the 318 HCEs are individual in any of the five query genomes.
