# Supplementary material for: Structural Relationships between Highly Conserved Elements and Genes in Vertebrate Genomes
Source: PLoS One. 2008 Nov 14;3(11):e3727. doi: 10.1371/journal.pone.0003727 (PMC2579482; doi:10.1371/journal.pone.0003727)
Supplement: Table S4 — The number of cases of HGLBs interlaced in the human genome but located on different chromosomes in other species. (0.03 MB DOC) [file pone.0003727.s008.doc]

| Species | Number of cases |
| --- | --- |
| Zebrafish | 8 |
| Tetraodon | 4 |
| Zebrafish, Tetraodon | 5 |
| Mouse, Rat, Zebrafish, Tetradon | 1 |
| Rat, Zebrafish, Tetraodon | 1 |
| Chicken, Zebrafish, Tetraodon | 1 |
| Chicken, Zebrafish | 1 |
| Rat | 1 |
| Total | 22 |
