# Supplementary material for: Structural Relationships between Highly Conserved Elements and Genes in Vertebrate Genomes
Source: PLoS One. 2008 Nov 14;3(11):e3727. doi: 10.1371/journal.pone.0003727 (PMC2579482; doi:10.1371/journal.pone.0003727)
Supplement: Table S5 — The number of HGLBs involved in the intersections in different vertebrate genomes. (0.03 MB DOC) [file pone.0003727.s009.doc]

|  | Human | Mouse | Rat | Chicken | Zebrafish | Tetraodon |
| --- | --- | --- | --- | --- | --- | --- |
| Number | 54 | 55 | 56 | 67 | 73 | 70 |
