# Supplementary material for: Structural Relationships between Highly Conserved Elements and Genes in Vertebrate Genomes
Source: PLoS One. 2008 Nov 14;3(11):e3727. doi: 10.1371/journal.pone.0003727 (PMC2579482; doi:10.1371/journal.pone.0003727)
Supplement: Table S6 — The number of HGLBs overlapped with 4-way synteny blocks in the human genome. (0.04 MB DOC) [file pone.0003727.s010.doc]

| Run of SBs | HGLBs intersected with single SB | | HGLBs intersected with more than one SBs | | | | Total HGLBs intersected with SB/SBs |
| --- | --- | --- | --- | --- | --- | --- | --- |
|  | Number | Percentage (%) | Number | Percentage (%) | SBs are  located on the same chromosome across the 4 species | |  |
|  |  |  |  |  | Number | Percentage (%) |  |
| 100K | 30 | 37.5 | 50 | 62.5 | 33 | 66.0 | 80 |
| 200K | 34 | 41.9 | 47 | 58.1 | 30 | 63.8 | 81 |
| 300K | 35 | 43.2 | 46 | 56.8 | 30 | 65.2 | 81 |
| 4 gene | 32 | 39.3 | 52 | 61.9 | 35 | 67.3 | 84 |
| 7 gene | 33 | 39.3 | 51 | 60.7 | 34 | 66.7 | 84 |
| 10 gene | 33 | 39.3 | 51 | 60.7 | 34 | 66. 7 | 84 |
| 20 gene | 34 | 40.5 | 50 | 59.5 | 33 | 66.0 | 84 |

SBs: 4-way syteny blocks
