# Supplementary material for: Structural Relationships between Highly Conserved Elements and Genes in Vertebrate Genomes
Source: PLoS One. 2008 Nov 14;3(11):e3727. doi: 10.1371/journal.pone.0003727 (PMC2579482; doi:10.1371/journal.pone.0003727)
Supplement: Table S7 — Sizes of HGLBs and 4-way syteny blocks in the human genome (Kb). (0.03 MB DOC) [file pone.0003727.s011.doc]

|  | | Min | Median | Mean | max |
| --- | --- | --- | --- | --- | --- |
| HGLBs | | 61 | 7,014 | 19,893 | 82,768 |
| 4-way syteny blocks | 100K | 101 | 989 | 2,613 | 43,636 |
|  | 200K | 218 | 1,641 | 3,450 | 43,636 |
|  | 300K | 325 | 2,419 | 3,948 | 43,636 |
|  | 4 gene | 23 | 1,067 | 2,826 | 49,242 |
|  | 7 gene | 23 | 1,286 | 3,085 | 49,242 |
|  | 10 gene | 23 | 1,391 | 3,245 | 49,242 |
|  | 20 gene | 23 | 1,717 | 3,683 | 49,242 |
