# Supplementary material for: Structural Relationships between Highly Conserved Elements and Genes in Vertebrate Genomes
Source: PLoS One. 2008 Nov 14;3(11):e3727. doi: 10.1371/journal.pone.0003727 (PMC2579482; doi:10.1371/journal.pone.0003727)
Supplement: Table S8 — Absolute relative distance differences (|RDD|s) of HCE-HCE pairs and HCE-gene pairs. (0.04 MB DOC) [file pone.0003727.s012.doc]

|  | | HCE-HCE | | |RDD| of HCE-gene | | | P value | | |
| --- | --- | --- | --- | --- | --- | --- | --- | --- | --- |
|  |  | \|RDD\| | Number | A (0Mb,1Mb] | B (1Mb,5Mb] | C (5Mb,~] | P(A,B) | P(A,C) | P(B,C) |
| Mouse | Median | 0.069 | 385 | 0.172 | 0.176 | 0.173 | 0.7325 | 0.3318 | 0.2023 |
|  | Mean | 0.123 |  | 0.207 | 0.189 | 0.372 |  |  |  |
| Rat | Median | 0.068 | 384 | 0.146 | 0.129 | 0.148 | 7.9e-05 | 5.9e-05 | 2.2e-16 |
|  | Mean | 0.126 |  | 0.185 | 0.154 | 0.402 |  |  |  |
| Chicken | Median | 0.314 | 387 | 0.667 | 0.859 | 0.895 | 4.4e-13 | 2.2e-16 | 0.0003 |
|  | Mean | 0.418 |  | 0.709 | 0.862 | 0.983 |  |  |  |
| Zebrafish | Median | 0.553 | 383 | 0.887 | 0.712 | 1.071 | 0.0016 | 0.5179 | 2.4e-05 |
|  | Mean | 0.628 |  | 0.977 | 0.883 | 1.016 |  |  |  |
| Tetraodon | Median | 1.060 | 387 | 1.458 | 1.596 | 1.606 | 0.0401 | 3.4e-09 | 4.8e-10 |
|  | Mean | 0.943 |  | 1.367 | 1.322 | 1.410 |  |  |  |
| Number of HCE-gene pairs | | | | 495 | 884 | 1526 |  |  |  |

P(X,Y) denotes the significance level (Wilcoxon’s test) for a comparison between sets of X and Y within different ranges of distance in the human genome.
