# Supplementary material for: Structural Relationships between Highly Conserved Elements and Genes in Vertebrate Genomes
Source: PLoS One. 2008 Nov 14;3(11):e3727. doi: 10.1371/journal.pone.0003727 (PMC2579482; doi:10.1371/journal.pone.0003727)
Supplement: Table S9 — Difference of |RDD| values for different pair wise elements. (0.03 MB DOC) [file pone.0003727.s013.doc]

|  | Human-Mouse | Human-Rat | Human-Chicken | Human-Zebrafish | Human-Tetraodon |
| --- | --- | --- | --- | --- | --- |
| P value  (HCE-HCE, HCE-gene) | 2.2e-16 | 2.2e-16 | 2.2e-16 | 2.2e-16 | 2.2e-16 |
| P value  (gene-gene, HCE-gene) | 2.2e-16 | 2.2e-16 | 2.2e-16 | 0.0405 | 2.2e-16 |
| P value  (exon-exon, HCE-gene) | 2.2e-16 | 2.2e-16 | 2.2e-16 | 0.0025 | 2.2e-16 |

Two samples Wilcoxon test was used to test the difference of |RDD| values between different pair wise elements
