# Supplementary material for: Structural Relationships between Highly Conserved Elements and Genes in Vertebrate Genomes
Source: PLoS One. 2008 Nov 14;3(11):e3727. doi: 10.1371/journal.pone.0003727 (PMC2579482; doi:10.1371/journal.pone.0003727)
Supplement: Table S10 — Overlapping between HCE blocks and CNE/UCR clusters. (0.06 MB DOC) [file pone.0003727.s014.doc]

**A:** Number of HCE blocks with X:Y overlapping pattern with CNE/UCR clusters, where X:Y refer to X HCE blocks overlapping with Y CNE/UCR clusters.

|  | | CNE cluster | | UCR cluster | |
| --- | --- | --- | --- | --- | --- |
|  |  | Y = 1 | Y > 1 | Y = 1 | Y > 1 |
| HCE block | X = 1 | 28 | 11 | 28 | 11 |
|  | X > 1 | 8 | 9 | 4 | 9 |

Based on the human genomic position, some HCE blocks are intersected with CNE/UCR clusters, which are defined by two independent works of Sandelin et al. [1] and Woolfe et al. [2]. Some CNE and UCR clusters are overlapped with more than one HCE block, which are located on different chromosomes in at least one query genome. A total of 56 HCE blocks are intersected with CNE clusters and 52 with UCR clusters respectively.

**B:** CNE clusters intersected with more than one HCE blocks

| Genomic Position of CNE cluster (hg18) | Number of HCE blocks intersected | Species in which the HCE blocks are located on different chromosomes |
| --- | --- | --- |
| chr1:24846125-25266721 | 2 | Zebrafish, Tetraodon |
| chr1:63120602-63687552 | 2 | Zebrafish |
| chr10:102362695-102969368 | 2 | Tetraodon |
| chr13:70936610-72123522 | 2 | Zebrafish |
| chr15:57984698-58605048 | 2 | Zebrafish, Tetraodon |
| chr15:64781543-64861173 | 2 | Tetraodon |
| chr15:65474304-68049198 | 4 | Zebrafish, Tetraodon |
| chr18:70477992-72060504 | 2 | Tetraodon |
| chr2:58000565-63122245 | 2 | Zebrafish |
| chr4:111774990-112889061 | 2 | Zebrafish, Tetraodon |
| chr4:124544185-124973309 | 2 | Zebrafish, Tetraodon |
| chr4:145497136-145864319 | 3 | Mouse, Rat, Zebrafish, Tetraodon |
| chr4:147435102-148177958 | 3 | Mouse, Rat, Zebrafish, Tetraodon |
| chr4:151493386-151821151 | 2 | Zebrafish, Tetraodon |

**C:** UCR clusters intersected with more than one HCE blocks

| Genomic Position of UCR cluster (hg18) | Number of HCE blocks intersected | Species in which the HCE blocks are located on different chromosomes |
| --- | --- | --- |
| chr1:62714881-64014881 | 2 | Zebrafish |
| chr10:101958765-104458765 | 2 | Tetraodon |
| chr13:70161365-72461365 | 2 | Zebrafish |
| chr15:57608155-59008155 | 2 | Zebrafish, Tetraodon |
| chr15:64508155-66308155 | 4 | Zebrafish, Tetraodon |
| chr15:67608155-68911129 | 3 | Zebrafish, Tetraodon |
| chr18:70066007-72466007 | 2 | Tetraodon |
| chr2:44395795-45395796 | 2 | Zebrafish |
| chr2:62595796-63595796 | 2 | Zebrafish |
| chr4:111326245-113326245 | 2 | Zebrafish, Tetraodon |
| chr4:140853264-141753264 | 2 | Zebrafish, Tetraodon |
| chr4:146953264-148653264 | 3 | Mouse, Rat, Zebrafish, Tetraodon |
| chr4:151253264-152253264 | 2 | Zebrafish, Tetraodon |

1. Sandelin A, Bailey P, Bruce S, Engstrom PG, Klos JM, et al. (2004) Arrays of ultraconserved non-coding regions span the loci of key developmental genes in vertebrate genomes. BMC Genomics 5: 99.

2. Woolfe A, Goodson M, Goode DK, Snell P, McEwen GK, et al. (2005) Highly conserved non-coding sequences are associated with vertebrate development. PLoS Biol 3: e7.
