# Supplementary material for: Structural Relationships between Highly Conserved Elements and Genes in Vertebrate Genomes
Source: PLoS One. 2008 Nov 14;3(11):e3727. doi: 10.1371/journal.pone.0003727 (PMC2579482; doi:10.1371/journal.pone.0003727)
Supplement: Table S11 — Summary statistics of HCE-gene pairs in the pair-wise comparisons between human and three non-mammal genomes. (0.04 MB DOC) [file pone.0003727.s015.doc]

|  | | Human-Chicken | Human-Zebrafish | Human-Tetraodon |
| --- | --- | --- | --- | --- |
| Number of HGLBs | | 75 | 190 | 126 |
| Number of HGLBs harboring more than one 4-way synteny blocks | | 74 | 190 | 126 |
| Percentage of associated HCEs (%) | | 99.5 | 99.1 | 99.8 |
| Number of HCEs for each HCE blocks | Median | 34 | 5 | 4 |
|  | Mean | 71 | 10 | 14 |
| Genes | Number | 8277 | 3319 | 5842 |
| Number of genes for each gene blocks | Median | 89 | 11 | 36 |
|  | Mean | 110 | 17 | 46 |
| CNE clusters intersected with more than one HCE blocks (%) | Number of CNE clusters | 40 | 107 | 46 |
|  | Number of HCEs | 5058 | 1759 | 1680 |
| UCR clusters intersected with more than one HCE blocks (%) | Number of UCR clusters | 28 | 90 | 46 |
|  | Number of HCEs | 5230 | 1825 | 1698 |

For pair-wise comparisons between human and three non-mammal genomes, more than ninety-nine percent of HCEs are found to be linked with set of gene/genes. A portion of previously reported HCE clusters (CNEs [1] and UCRs [2]), are also found divided into several HCE blocks.

1. Woolfe A, Goodson M, Goode DK, Snell P, McEwen GK, et al. (2005) Highly conserved non-coding sequences are associated with vertebrate development. PLoS Biol 3: e7.

2. Sandelin A, Bailey P, Bruce S, Engstrom PG, Klos JM, et al. (2004) Arrays of ultraconserved non-coding regions span the loci of key developmental genes in vertebrate genomes. BMC Genomics 5: 99.
