# Supplementary material for: Structural Relationships between Highly Conserved Elements and Genes in Vertebrate Genomes
Source: PLoS One. 2008 Nov 14;3(11):e3727. doi: 10.1371/journal.pone.0003727 (PMC2579482; doi:10.1371/journal.pone.0003727)
Supplement: Table S12 — Percentage of genes associated with HCEs over total genes in the overall genomic region covered by HGLBs, and the number of intervening “genes” in between each HCE-gene pair in the human genome. (0.03 MB DOC) [file pone.0003727.s016.doc]

|  | Min | Median | Mean | Max |
| --- | --- | --- | --- | --- |
| Total number of genes in the associated genomic region | 1 | 63 | 161 | 1054 |
| Percentage of genes associated with HCE | 0.4 | 4.5 | 13.1 | 100 |
| Number of intervening genes | 0 | 64 | 132 | 1026 |

The number of genes overlapping in their genomic loci was counted as one.
