# Supplementary figures and images for: Structural Relationships between Highly Conserved Elements and Genes in Vertebrate Genomes
Source: PLoS One. 2008 Nov 14;3(11):e3727. doi: 10.1371/journal.pone.0003727 (PMC2579482; doi:10.1371/journal.pone.0003727)

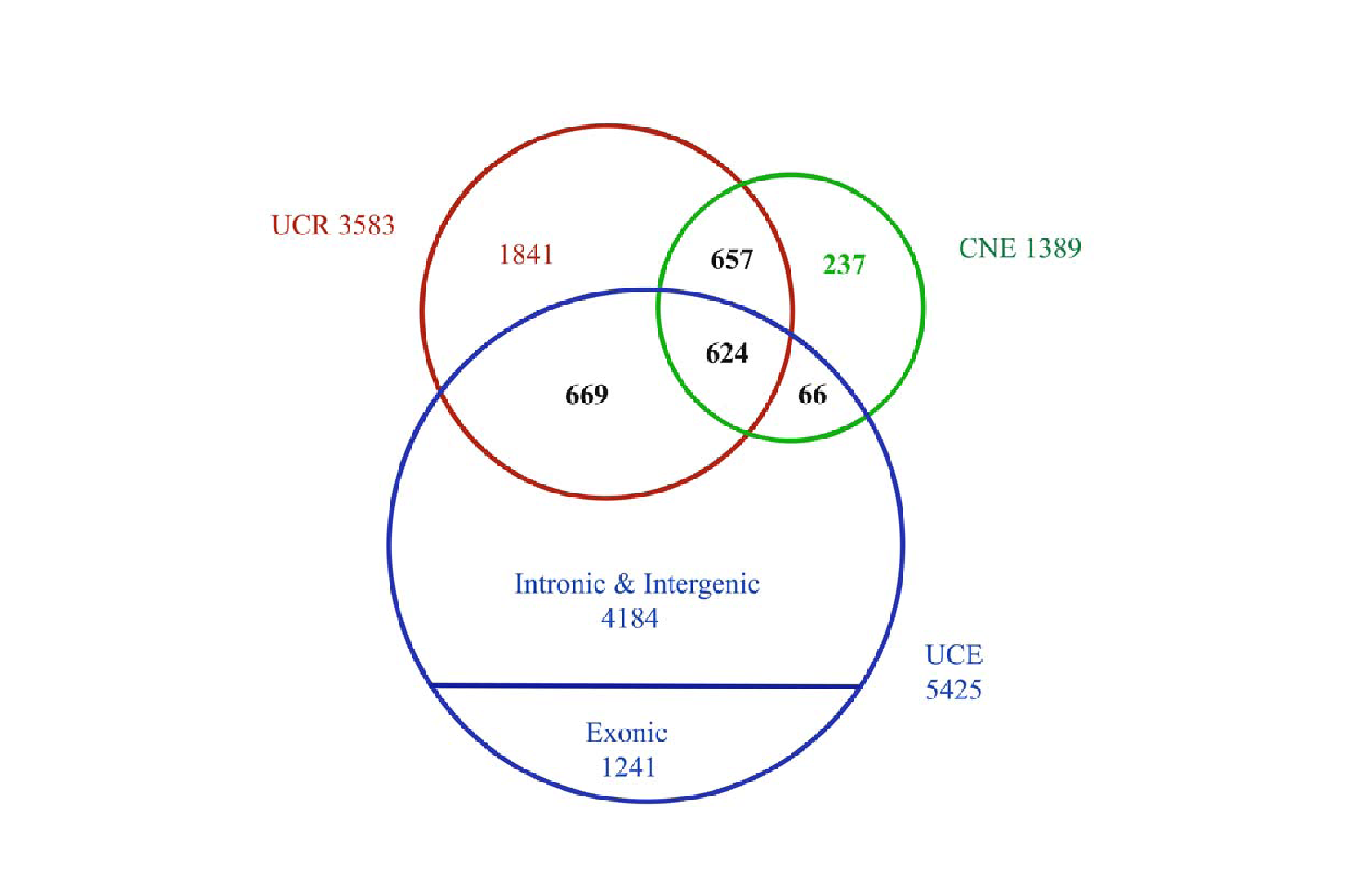

Supplement: Figure S1 — Overlapping between HCEs from different data sets. The figure shows UCRs (red), CNEs (green) and UCEs (blue) with at least partial (more than 1 bp) overlapping in the human genome. The data from the three studies were derived from three different versions of the human genome sequence, and had to be mapped onto a common version for comparison. Thus, the number of total HCE for the two first datasets differs slightly from the figures published by the original studies. (0.52 MB TIF) [file pone.0003727.s017.tif]

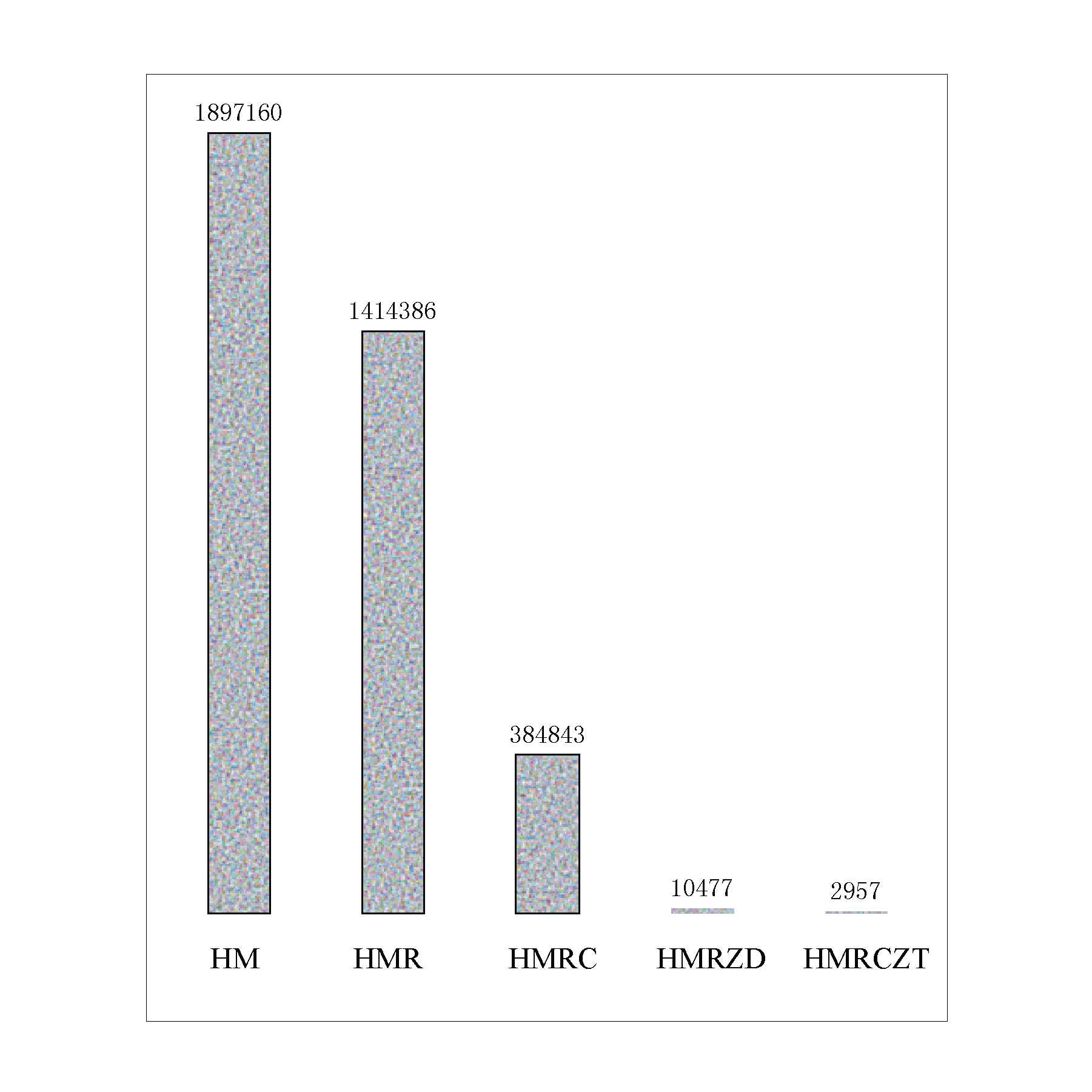

Supplement: Figure S3 — Number of conserved HCE-gene pairs at different conservation level. (HM: in the human-mouse comparison; HMR: in the human-mouse-rat comparison etc. H stands for human, M for mouse, R for rat, C for chicken, Z for zebrafish and T for tetraodon) (1.26 MB TIF) [file pone.0003727.s019.tif]

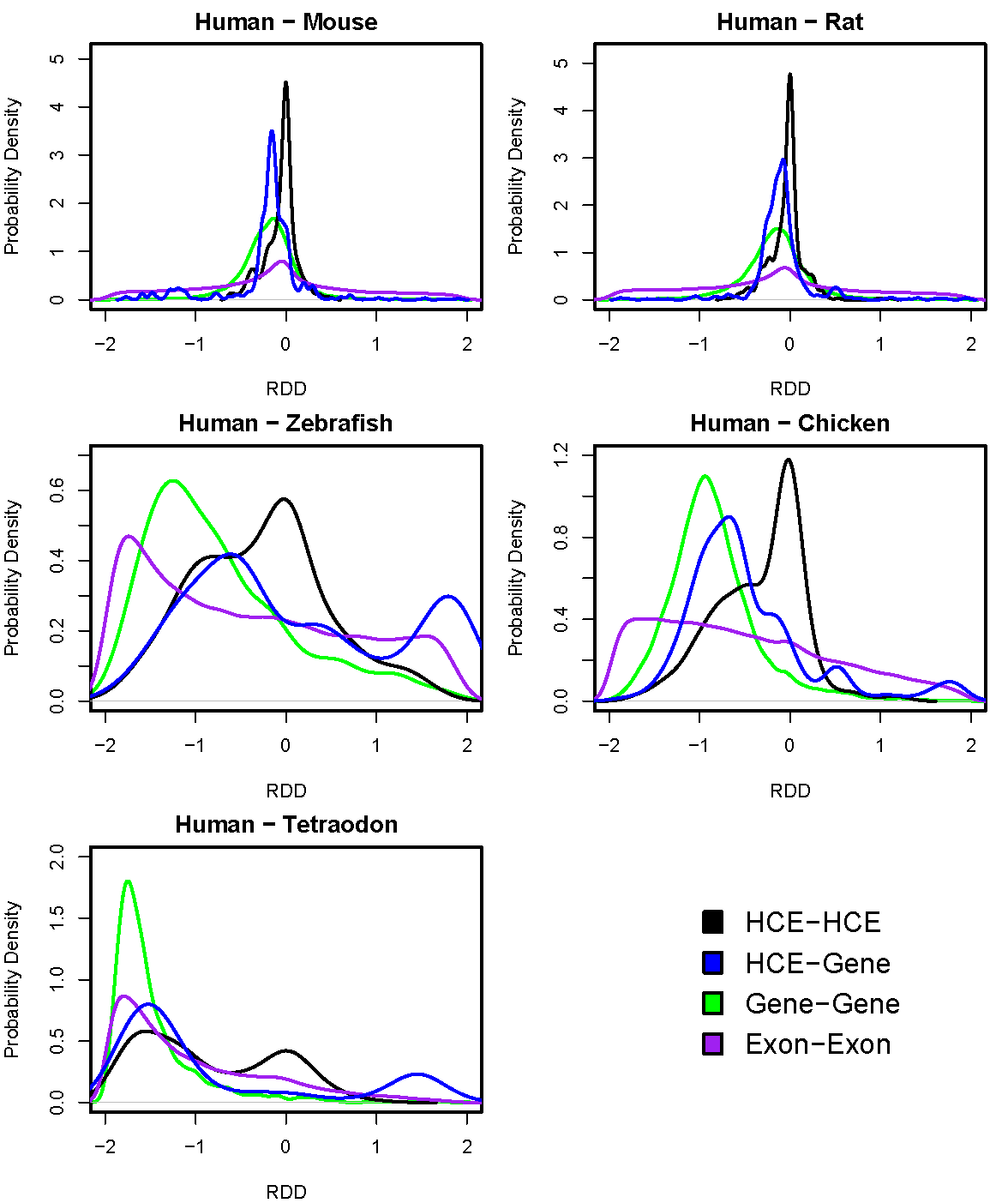

Supplement: Figure S6 — RDD distribution of four sets of data (HCE-HCE, HCE-gene, Gene-Gene and Exon-Exon). (0.16 MB TIF) [file pone.0003727.s022.tif]

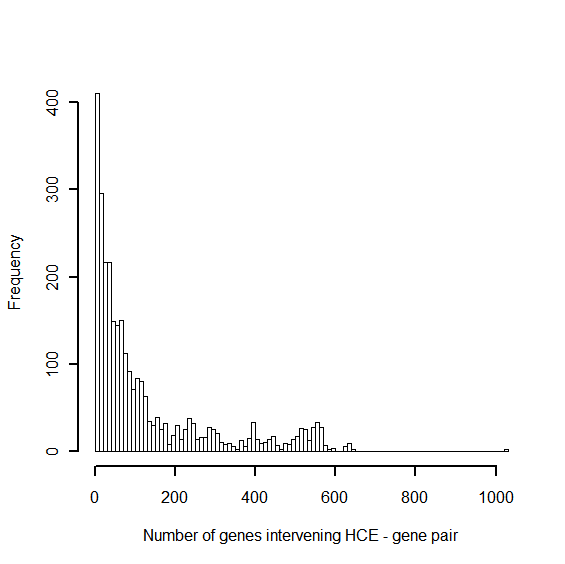

Supplement: Figure S8 — Histogram of the number of genes intervening HCE-gene pairs based on the human genome annotation. The number of genes overlapping in their genomic loci was counted as one. (0.96 MB TIF) [file pone.0003727.s024.tif]
